# Supplementary material for: Mechanisms governing the pioneering and redistribution capabilities of the non-classical pioneer PU.1
Source: Nat Commun. 2020 Jan 21;11:402. doi: 10.1038/s41467-019-13960-2 (PMC6972792; doi:10.1038/s41467-019-13960-2)
Supplement: Supplementary file 7 — Source data [file 41467_2019_13960_MOESM7_ESM.zip › Source_Data/FigureS3/FigureS3H_MotifScanOutput/homerResults/motif3.similar.html]

motif3

## Information for motif3

A
C
T
G
G
C
T
A
A
C
T
G
A
T
C
G
A
C
G
T
C
G
A
T
C
G
A
T
  
Reverse Opposite:  

C
G
T
A
C
G
T
A
G
T
C
A
A
T
G
C
T
G
A
C
C
G
A
T
T
G
A
C
  

|  |  |
| --- | --- |
| p-value: | 1e-190 |
| log p-value: | -4.386e+02 |
| Information Content per bp: | 1.724 |
| Number of Target Sequences with motif | 1803.0 |
| Percentage of Target Sequences with motif | 46.64% |
| Number of Background Sequences with motif | 10659.8 |
| Percentage of Background Sequences with motif | 23.80% |
| Average Position of motif in Targets | 276.8 +/- 280.6bp |
| Average Position of motif in Background | 209.9 +/- 140.0bp |
| Strand Bias (log2 ratio + to - strand density) | 0.1 |
| Multiplicity (# of sites on avg that occur together) | 1.20 |
| Motif File: | file (matrix) reverse opposite |

### Similar de novo motifs found

|  |  |  |  |  |  |  |  |
| --- | --- | --- | --- | --- | --- | --- | --- |
| Rank | Match Score | Redundant Motif | P-value | log P-value | % of Targets | % of Background | Motif file |
| 1 | 0.779 | G T A C C A G T A C T G T G A C A C T G A T C G A G C T A C G T C A G T | 1e-85 | -196.222739 | 37.45% | 22.75% | motif file (matrix) |
| 2 | 0.775 | A C G T A C G T A T C G A T C G A C G T A C G T C G A T A G T C | 1e-21 | -48.460981 | 15.26% | 10.10% | motif file (matrix) |
